# Beyond the Surface: Duodenal‐Type Follicular Lymphoma Diagnosed Despite Normal‐Appearing Duodenal Mucosa and Absence of Focal Duodenal FDG Uptake

**DOI:** 10.1155/crgm/1030883

**Published:** 2026-06-16

**Authors:** Zachary Vinton, Carter Schulz, William McGuire, Safina Hafeez, Sarah Malik

**Affiliations:** ^1^ Department of Internal Medicine, University of Nebraska Medical Center, Omaha, Nebraska, USA, unmc.edu; ^2^ Department of Pathology, Microbiology, and Immunology, University of Nebraska Medical Center, Omaha, Nebraska, USA, unmc.edu; ^3^ Department of Internal Medicine, Division of Gastroenterology and Hepatology, University of Nebraska Medical Center, Omaha, Nebraska, USA, unmc.edu

## Abstract

Duodenal‐type follicular lymphoma (DTFL) is an uncommon and often indolent extranodal lymphoma that may be difficult to recognize when characteristic endoscopic or imaging findings are absent. We report a 60‐year‐old man evaluated for progressive fatigue, epigastric pain, and iron deficiency whose esophagogastroduodenoscopy showed normal‐appearing duodenal mucosa. Random duodenal biopsies revealed follicular lymphoma with morphology and immunophenotype consistent with follicular lymphoma, duodenal type, in the absence of systemic follicular lymphoma. Staging positron emission tomography/computed tomography (PET/CT) showed no focal duodenal fluorodeoxyglucose uptake but demonstrated equivocal low‐level uptake in mildly prominent mesenteric lymph nodes. Given unclear staging and persistent gastrointestinal symptoms affecting quality of life, the patient was treated with rituximab monotherapy. Follow‐up PET/CT findings were consistent with complete metabolic response, and repeat duodenal biopsies showed no residual lymphoma. This case highlights the diagnostic value of random duodenal biopsy in unexplained iron deficiency and emphasizes the staging complexity of DTFL when PET/CT findings are equivocal.

## 1. Introduction

Duodenal‐type follicular lymphoma (DTFL) is an uncommon extranodal variant of follicular lymphoma, estimated to account for 1.0%–6.0% of gastrointestinal follicular lymphoma cases [1]. DTFL is typically slow‐growing and characterized by low tumor burden with a strong predilection for mucosal involvement of the proximal duodenum [2]. Although prognosis is generally favorable, recognition can be challenging because clinical manifestations are often nonspecific and characteristic endoscopic abnormalities may be subtle or absent [3].

While many patients with DTFL are asymptomatic, clinically significant disease may manifest with chronic abdominal discomfort, iron deficiency anemia, fatigue, or malabsorptive symptoms that impair quality of life [3]. Endoscopic visualization and cross‐sectional imaging are commonly used to guide targeted biopsy and staging of gastrointestinal lymphomas; however, these modalities may have limited sensitivity in detecting low‐grade, mucosa‐limited disease [4]. We present a case of DTFL diagnosed exclusively through random duodenal biopsies during evaluation for iron deficiency, highlighting important diagnostic and staging considerations.

## 2. Case Presentation

A 60‐year‐old man presented to his primary care physician with several months of progressive fatigue, exertional dyspnea, and restless leg symptoms. His medical history was notable for non‐Hodgkin lymphoma diagnosed 34 years prior. Records clarifying the original lymphoma subtype, stage, treatment regimen, and pathologic features were unavailable. The patient recalled receiving six cycles of chemotherapy without radiation or surgery. Posttreatment imaging reportedly demonstrated clinical remission, and he subsequently underwent active surveillance without known recurrent disease.

He also reported chronic postprandial heartburn for which he took a daily proton pump inhibitor and intermittent mild hematochezia attributed to hemorrhoids, but denied melena, unintentional weight loss, fevers, or night sweats. He denied nonsteroidal anti‐inflammatory drug use, tobacco use, or alcohol consumption. A screening colonoscopy performed 10 years earlier was unremarkable.

Initial laboratory evaluation demonstrated iron deficiency with borderline normocytic anemia, including ferritin 6 ng/mL, hemoglobin 13.2 g/dL, and mean corpuscular volume 81.5 fL. Oral iron supplementation was started, but was not tolerated due to worsening of postprandial abdominal pain. Due to his iron deficiency and his epigastric pain not improving with chronic proton pump inhibitor therapy, he was referred for bidirectional endoscopic evaluation.

**FIGURE 1:**
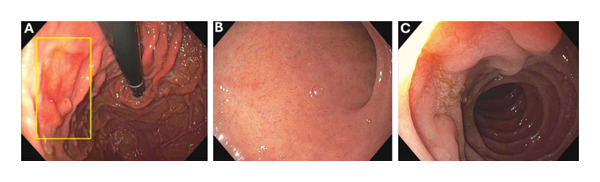
Endoscopic findings from bidirectional endoscopy. (A) Fifteen‐millimeter ulcer in the gastric fundus. (B) First portion of the duodenum demonstrating normal‐appearing mucosa without nodularity or ulceration. (C) Second portion of the duodenum demonstrating grossly normal mucosa.

Two months later, an esophagogastroduodenoscopy (EGD) demonstrated a single 15‐mm superficial gastric ulcer‐appearing lesion in the fundus, multiple 3‐ to 5‐mm sessile fundic gland polyps in the gastric body, and mild patchy gastric erythema (Figure 1).

The remainder of the stomach was normal. The duodenum appeared grossly normal without nodularity, ulceration, polypoid change, or mucosal irregularity; however, random biopsies were obtained routinely for iron deficiency to rule out celiac disease. Colonoscopy to the terminal ileum was complete with adequate bowel preparation (Boston Bowel Prep Score of 9) and showed diverticulosis without noted internal hemorrhoids. A 2‐mm sessile cecal polyp was removed. Otherwise, the examined terminal ileum and colon appeared normal with no mucosal abnormalities.

**FIGURE 2:**
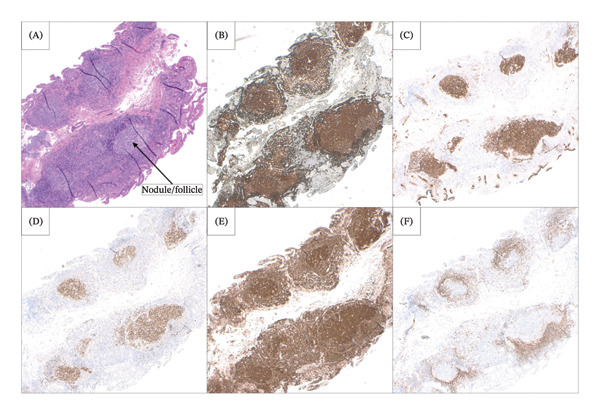
Histologic and immunohistochemical findings supporting follicular lymphoma, duodenal type. Random duodenal biopsies included five intestinal mucosal fragments, two of which were involved in follicular lymphoma. Hematoxylin and eosin sections demonstrated intestinal mucosa with an atypical lymphoid infiltrate in a nodular/follicular pattern composed predominantly of small angulated centrocytes, without sheets of large cells (A). The neoplastic cells were positive for CD20 (B), CD10 (C), BCL6 (D), BCL2 (E), and CD23 (F). CD5 and CyclinD1 were negative.

Random duodenal biopsies included five intestinal mucosal fragments, two of which were involved in follicular lymphoma. Hematoxylin and eosin sections demonstrated intestinal mucosa with an atypical lymphoid infiltrate in a nodular/follicular pattern composed predominantly of small angulated centrocytes, without sheets of large cells (Figure 2A).

Immunohistochemistry is summarized in Table 1 and demonstrated neoplastic follicles positive for CD20 (Figure 2B), CD10 (Figure 2C), and BCL‐6 (Figure 2D), with co‐expression of BCL‐2 (Figure 2E). CD23 highlighted follicular dendritic cell meshwork displaced to the periphery of the neoplastic follicles, producing a hollow meshwork appearance (Figure 2F). Ki‐67 demonstrated a low proliferative index. *IGH::BCL2* rearrangement testing by fluorescence in situ hybridization was not performed because the diagnosis was established by morphology and immunophenotype. Overall, the findings support follicular lymphoma. In the absence of systemic follicular lymphoma, the findings are consistent with follicular lymphoma, duodenal type.

**TABLE 1:** Immunohistochemical findings supporting follicular lymphoma, duodenal type.

Marker/ancillary study	Result	Diagnostic significance
CD20	Positive in B cells within and outside nodules	Supports mature B‐cell lineage
CD10	Positive in follicles, with scattered positivity outside follicles	Supports germinal‐center B‐cell phenotype
BCL‐2	Co‐expressed in neoplastic follicles	Supports follicular lymphoma over reactive germinal centers
BCL‐6	Positive in follicles	Supports germinal‐center B‐cell phenotype
CD3	Highlights background T cells	Supports the background T‐cell population
CD5	Negative in neoplastic follicles	Argues against CD5‐positive B‐cell lymphoma
Cyclin D1	Negative	Argues against mantle cell lymphoma
Ki‐67	< 10% proliferative index	Supports low proliferative activity
CD23	Peripheral/hollow follicular dendritic cell meshworks	Supports abnormal/neoplastic follicular architecture
*IGH::BCL2* rearrangement by FISH	Not performed	Not required for diagnosis because morphology and immunophenotype were diagnostic

Gastric biopsies showed antral and body‐type mucosa with mild reactive gastropathy and were negative for *Helicobacter pylori*. Biopsies from the gastric ulcer demonstrated fundic gland polyps with focal iron deposition, suggestive of iron pill gastritis. Cecal polypectomy showed benign colonic mucosa with a prominent lymphoid aggregate without immunophenotypic evidence of follicular lymphoma. Celiac serologies were not obtained; however, duodenal biopsies did not show histologic features consistent with celiac disease.

The patient was referred to oncology for staging. Positron emission tomography/computed tomography (PET/CT) demonstrated low‐level uptake in mildly prominent central mesenteric lymph nodes measuring up to 0.9 × 1.0 cm with a maximum standardized uptake value (SUV) of 2.4, similar to blood pool activity and corresponding to Deauville score 2. No discrete FDG‐avid duodenal lesion was identified to correspond to the biopsy‐proven follicular lymphoma. PET/CT also showed asymmetric uptake in the anterior right prostate, which was followed by urology in the context of known prostatitis and benign prostatic hyperplasia.

Peripheral blood smear and right iliac crest bone marrow aspiration, clot, and core biopsy were performed. Bone marrow pathology demonstrated cellular marrow with trilineage hematopoiesis and no morphologic evidence of lymphoma. Flow cytometry showed no abnormal or neoplastic lymphoid population with polyclonal B‐cells and plasma cells. Iron staining showed decreased uptake graded as 2/6 without ring sideroblasts. The patient received intravenous iron supplementation, with subsequent normalization of hemoglobin, ferritin, and transferrin saturation.

Given the solitary duodenal biopsy finding, absence of significantly enlarged lymphadenopathy, low‐level mesenteric nodal avidity, and negative bone marrow evaluation, oncology documented Stage IA versus Stage IVA follicular lymphoma, duodenal type. After discussion about a watch‐and‐wait strategy versus systemic treatment, rituximab monotherapy was selected because of concern for possible advanced‐stage disease, persistent upper gastrointestinal symptoms, and shared decision‐making. The patient received four doses of rituximab weekly, but did experience recurrent infusion‐related reactions, including anxiety with chest discomfort and itching of the eyes, ears, and abdomen. End‐of‐treatment PET/CT demonstrated no new or worsening hypermetabolic lymphadenopathy or lymphomatous involvement, with minimal residual mesenteric activity below blood pool background, consistent with complete metabolic response. Follow‐up EGD 3 months post‐rituximab demonstrated no residual lymphoma on multiple biopsies, including seven fragments from the first part of the duodenum and 10 fragments of the second part of the duodenum. The patient’s clinical course, diagnostic evaluation, treatment, and follow‐up are summarized in Table 2.

**TABLE 2:** Timeline of symptoms, diagnostic evaluation, treatment, and follow‐up.

Date	Event	Details and key findings
1990	Remote NHL diagnosis	Six cycles of chemotherapy without radiation or surgery; subtype, stage, and exact treatment regimen unavailable
Sept. 2024	Primary care evaluation for restless leg symptoms and fatigue	Ferritin 6 ng/mL; hemoglobin 13.2 g/dL; MCV 81.5 fL
Dec. 2024	EGD and colonoscopy	EGD showed a 15‐mm superficial gastric ulcer, fundic gland polyps, mild gastric erythema, and a normal‐appearing duodenum; random duodenal biopsies showed Grade 1‐2 follicular lymphoma involving three duodenal mucosal biopsy fragments; gastric ulcer biopsy showed focal iron deposition suggestive of iron pill gastritis
Dec. 2024	Staging PET/CT	No discrete FDG‐avid duodenal lesion; mildly prominent central mesenteric lymph nodes up to 0.9 × 1.0 cm, SUV max 2.4, Deauville 2; no disease above the diaphragm
Jan. 2025	Bone marrow biopsy and flow cytometry	Cellular marrow with trilineage hematopoiesis; no morphologic evidence of lymphoma; flow cytometry without abnormal or neoplastic lymphoid population
Jan. 2025	Oncology consultation	Oncology documented Stage IA versus Stage IVA follicular lymphoma, duodenal type; staging uncertainty due to isolated duodenal biopsy finding and equivocal mesenteric nodal uptake; active surveillance versus systemic therapy discussed
Jan.–Feb. 2025	Rituximab treatment	Four planned once‐weekly doses of rituximab; course complicated by infusion‐related reactions
Jun. 2025	End‐of‐treatment PET/CT	No new or worsening hypermetabolic lymphadenopathy or lymphomatous involvement; minimal residual mesenteric activity below the blood pool; consistent with complete metabolic response
Aug. 2025	Follow‐up EGD	Resolution of gastric ulcer; normal‐appearing stomach and duodenum; seven random biopsies from D1 and 10 from D2 showed no histopathologic evidence of residual lymphoma
Dec. 2025	Repeat PET/CT	No evidence of residual or recurrent lymphoma

*Note:* EGD, esophagogastroduodenoscopy; FDG, fluorodeoxyglucose.

Abbreviations: DTFL, duodenal‐type follicular lymphoma; NHL, non‐Hodgkin lymphoma; PET/CT, positron emission tomography/computed tomography; SUV, standardized uptake value.

## 3. Discussion

This case highlights an uncommon diagnostic presentation of DTFL, in which the disease was identified exclusively through random duodenal biopsies obtained from normal‐appearing duodenal mucosa, without an endoscopic or radiographic correlation to guide targeted sampling. Although DTFL is typically an indolent extranodal lymphoma with low tumor burden and a predilection for mucosal involvement of the proximal small intestine, this case demonstrates how reliance on visual endoscopic findings alone may fail to detect clinically significant disease [2].

In contrast to most reported cases of DTFL, which describe characteristic endoscopic findings such as multiple small white nodular or polypoid lesions in the second portion of the duodenum, this patient’s duodenal mucosa was grossly normal despite histopathologic evidence of lymphoma [5]. Prior case reports have emphasized the diagnostic utility of targeted biopsy of visible lesions to establish diagnosis, whereas in this case, random biopsies were obtained during evaluation for iron deficiency in the absence of any macroscopic abnormality, highlighting an unusual diagnostic presentation [5–11]. This underscores an important limitation of reliance on visual endoscopic assessment alone when evaluating patients with unexplained iron deficiency anemia or persistent epigastric pain.

This case also illustrates the staging complexity of duodenal follicular lymphoma. Although the duodenal biopsy established follicular lymphoma, the extent of mucosal disease was uncertain because the diagnosis was made on random biopsies of normal‐appearing mucosa. PET/CT showed low‐level avidity in mildly prominent central mesenteric lymph nodes, but the nodes were not significantly enlarged, demonstrated uptake similar to blood pool, and were not biopsy‐proven. Bone marrow biopsy and flow cytometry showed no evidence of lymphoma. Accordingly, oncology considered staging difficult, documenting Stage IA versus Stage IVA follicular lymphoma, duodenal type. Under a GI‐specific Lugano framework, disease confined to the duodenum would be Stage I, whereas mesenteric nodal involvement, if determined to be lymphoma, would more likely represent abdominal nodal disease (Lugano Stage II2) rather than definitive disseminated extranodal Stage IV disease.

The patient’s remote history of non‐Hodgkin lymphoma introduces diagnostic uncertainty. Records from the original diagnosis were unavailable, including subtype, stage, treatment regimen, and pathology, preventing direct comparison with the current duodenal biopsy. Although the patient reported completing chemotherapy 34 years earlier with posttreatment imaging consistent with remission and no known recurrence during long‐term surveillance, late relapse of a prior follicular lymphoma or secondary gastrointestinal involvement by systemic follicular lymphoma cannot be definitively excluded. However, the prolonged remission interval, mucosa‐based duodenal involvement, negative bone marrow evaluation, equivocal low‐level mesenteric nodal uptake, and absence of definitive systemic disease on current staging support the interpretation of follicular lymphoma, duodenal type. This classification was based on the morphologic and immunophenotypic findings in the duodenal biopsy, interpreted in conjunction with clinical and radiologic staging that did not demonstrate definitive systemic follicular lymphoma.

The relationship between DTFL and the patient’s iron deficiency is uncertain. Iron deficiency prompted the endoscopic evaluation that led to diagnosis and was considered during treatment planning; however, alternative contributors were present, including a gastric ulcer with focal iron deposition suggestive of iron pill gastritis and reported intermittent hemorrhoidal bleeding (not observed endoscopically). The patient’s hemoglobin and iron indices improved after intravenous iron supplementation. Although anemia has been reported in patients with DTFL, it is not a defining feature of the disease, and this case should not be interpreted as establishing DTFL as the sole etiology of iron deficiency [3].

In addition, PET/CT findings in DTFL are variable. Although some lesions demonstrate focal FDG uptake, low‐volume mucosal disease may be below the spatial or metabolic resolution of PET/CT, as observed in this case [12]. PET/CT interpretation in the small bowel may also be limited by physiologic bowel activity, peristalsis, normal resident lymphoid tissue uptake, and granulomatous or inflammatory conditions [12]. In a series by Iwamuro et al., FDG uptake was identified in only 46% of patients with gastrointestinal follicular lymphoma, while PET/CT failed to detect known gastrointestinal involvement in a substantial proportion of cases [13]. Therefore, the absence of focal duodenal FDG uptake should not exclude mucosal follicular lymphoma when histologic suspicion remains.

Management strategies for DTFL range from active surveillance to systemic and/or radiation therapy, reflecting its typically indolent course and favorable prognosis [14]. Observation is frequently appropriate for asymptomatic patients with localized disease; however, treatment may be warranted based on the presence of symptom burden, advanced‐stage disease, or concern for ongoing complications [14]. Importantly, long‐term outcomes remain incompletely defined, as previous studies have included both small cohorts and limited patient follow‐up [1]. Chouhan et al. analyzed the Surveillance, Epidemiology, and End Results Registry (SEER) and observed a 5‐year overall survival of approximately 81% in small intestinal follicular lymphoma with increased mortality in patients aged 66 or older and Ann Arbor Stage III/IV disease [1]. With this in mind, rituximab monotherapy was selected in this case, given uncertain staging with potential mesenteric lymph node involvement and persistent epigastric pain despite proton pump inhibitor therapy, along with shared decision making.

In summary, this case illustrates the diagnostic, staging, and management complexities of DTFL. Clinicians should maintain a high index of suspicion for gastrointestinal lymphoma in patients presenting with subtle symptoms such as fatigue, dyspepsia, and iron deficiency; recognize the limitations of standard imaging modalities; and appreciate the diagnostic value of random duodenal biopsy even when mucosal abnormalities are not apparent.

## Funding

No funding was received for this project.

## Ethics Statement

Written informed consent was obtained from the patient for publication of this case report and accompanying images. This case report has been fully anonymized.

## Conflicts of Interest

The authors declare no conflicts of interest.

## Data Availability

The data that support the findings of this study are available on request from the corresponding author. The data are not publicly available due to privacy or ethical restrictions.
